# *Erratum:* Vol. 60, No. RR-1

**DOI:** 10.15585/mmwr.mm6626a7

**Published:** 2017-07-07

In the *Recommendations and Reports* “Antiviral Agents for the Treatment and Chemoprophylaxis of Influenza” (January 21, 2011, Vol. 60, No. RR-1, https://www.cdc.gov/mmwr/pdf/rr/rr6001.pdf), on page 8, in the first column, in the second paragraph, in the second sentence, the term “pneumonia” was used rather than “lower respiratory tract complications leading to antibiotic use.” The corrected sentence should read, “In a study that combined data from 10 clinical trials, the risk for **lower respiratory tract complications leading to antibiotic use** among those participants with laboratory-confirmed influenza receiving oseltamivir treatment was approximately 50% lower than among those persons receiving a placebo and 34% lower among patients at risk for complications (p<0.05 for both comparisons) (*22*).”

This correction does not change CDC’s influenza antiviral recommendations. A summary of current antiviral guidance is available at https://www.cdc.gov/flu/professionals/antivirals/summary-clinicians.htm.

